# Supplementary material for: Molecular characterisation of the acyltransferase-acyl carrier protein interface in a fungal highly reducing polyketide synthase
Source: RSC Chem Biol. 2025 Dec 9;7(2):233–9. doi: 10.1039/d5cb00278h (PMC12703375; doi:10.1039/d5cb00278h)
Supplement: CB-007-D5CB00278H-s001 [file CB-007-D5CB00278H-s001.pdf]

Supplementary Information (SI) for RSC Chemical Biology.
This journal is © The Royal Society of Chemistry 2025

Supporting Information

Molecular characterisation of the acyltransferase-acyl carrier protein interaction in a fungal highly reducing polyketide synthase.

Mia E. Foran, Y. T. Candace Ho[a], Józef R. Lewandowski[a] and Matthew Jenner[a]*

[a] Department of Chemistry, University of Warwick, Coventry, CV4 7AL, UK

* Corresponding author: Matthew Jenner (m.jenner@warwick.ac.uk)

1. Supplementary Figures

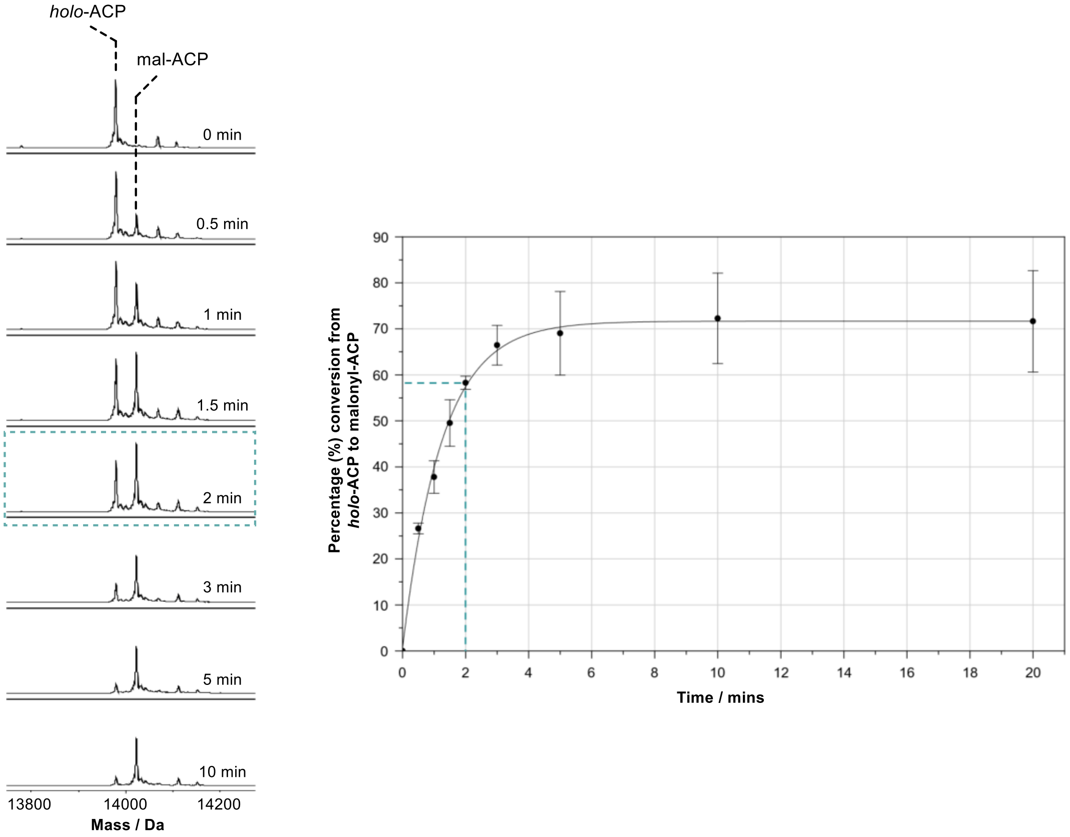

Supplementary Figure S1 | Time-course of SimG AT-catalysed malonyl transfer to SimG ACP. Stacked deconvoluted mass spectra (*left*) and plot (*right*) of the malonyl-transfer reaction between SimG AT and *holo*-SimG ACP domains over a 10 min time-course. The ratio between the *holo*-ACP and malonyl-ACP peaks was measured for each time point and plotted as percentage conversion. The 2-minute time point (indicated with a dashed line, 58.3 ± 1.44 %) was selected to assess the relative activities of the ACP domain X→Ala mutants, as it is situated in the linear region of the curve.

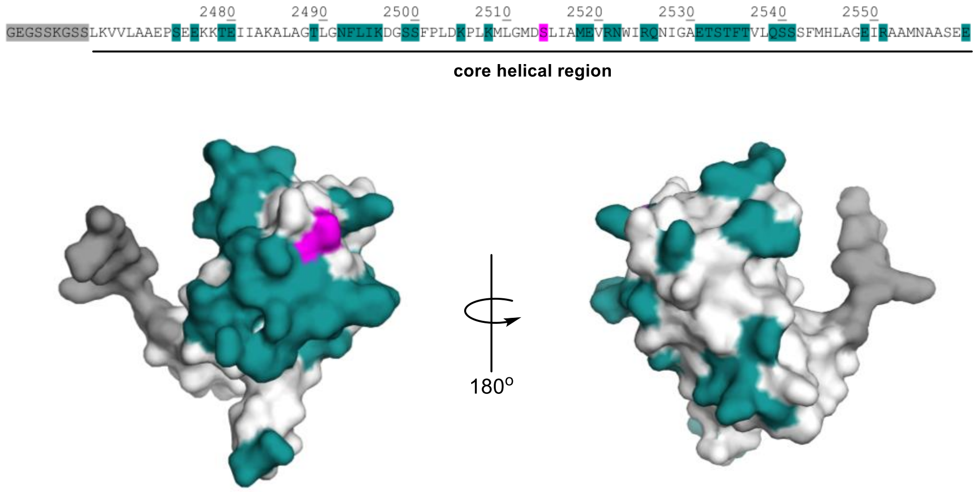

Supplementary Figure S2 | Coverage of SimG ACP domain with X→Ala mutations. Amino acid sequence (*top*) and AlphaFold 3 model (*bottom*) of the SimG ACP domain is shown. The core helical region is underlined and all X→Ala mutants are highlighted in teal. Approximately 49 % of the solvent exposed non-Ala/Gly residues from the helical core were covered by the scanning alanine mutagenesis approach. The Ppant attachment site, Ser2514, is highlighted in magenta.

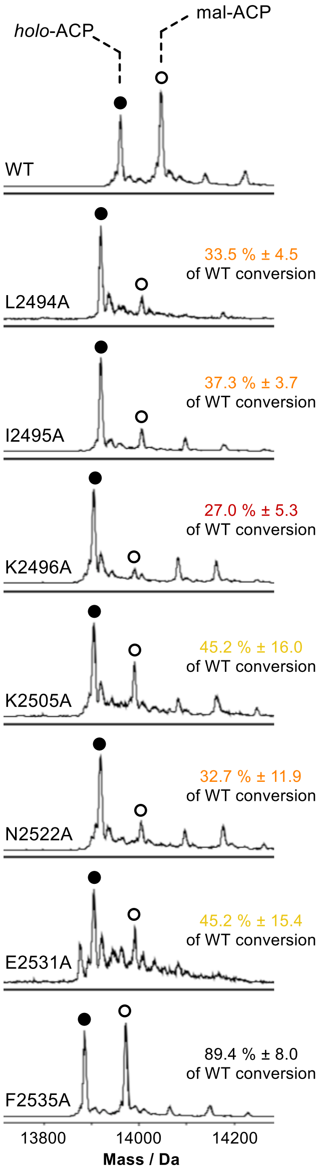

Supplementary Figure S3 | Example spectra of malonyl transfer reactions for SimG ACP domain X→Ala mutants. Deconvoluted mass spectra of SimG ACP domain (WT and selected X→Ala mutants) after 2 min malonyl transfer reaction with SimG AT domain. Values are reported relative to WT SimG ACP transfer (58.3 ± 1.44 %) and errors represent ± 1 standard deviation (1σ) from the mean, where *n* = 3. Activities relative to WT are highlighted in red (< 30 %), orange (30 - 40 %), yellow (40 - 50 %), and black (> 50 %). The F2523A mutant is displayed as an example of a mutation that obtained near-WT levels of acylation.

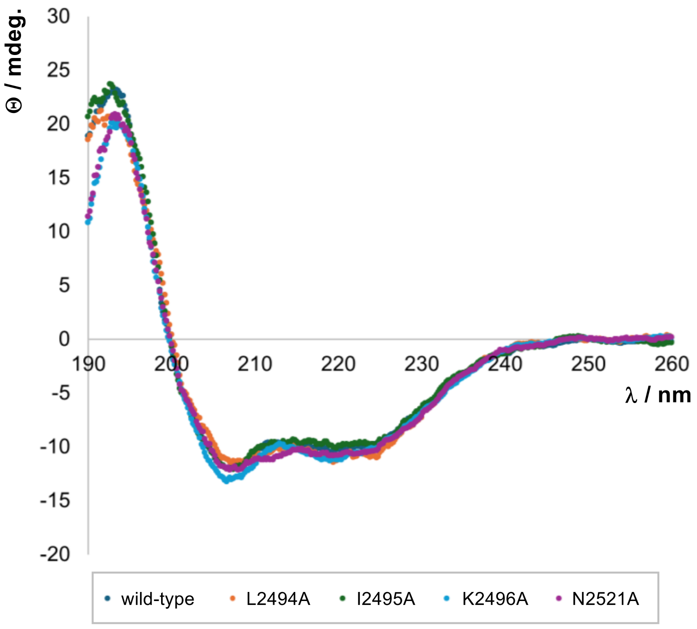

Supplementary Figure S4 | Circular dichroism spectroscopic analysis of SimG ACP X→Ala mutants. Overlaid CD spectra for wild-type SimG ACP domain and four alanine mutants which disrupted the interaction with SimG AT domain. The CD spectra obtained for each mutant are in agreement with the WT PksJ ACP4 domain, suggesting no loss / gain of secondary structure has occurred upon mutation.

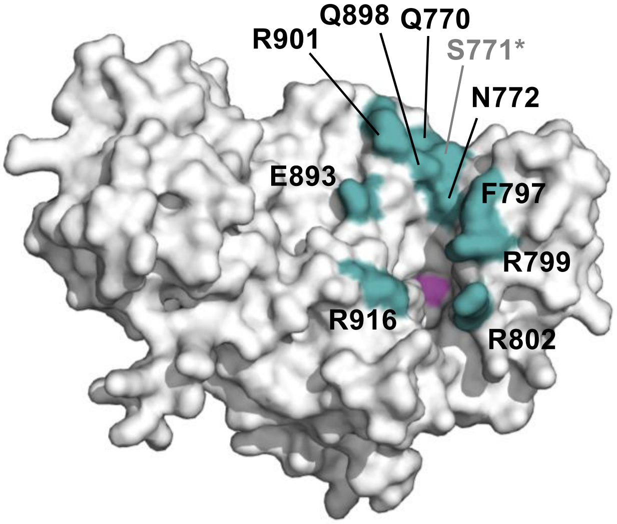

Supplementary Figure S5 | SimG AT domain X→Ala mutations. AlphaFold 3 model of SimG AT domain with residues surrounding the Ppant / CoA binding cleft highlighted in teal and labelled. All residues were mutated to Ala and yielded soluble protein. The S771A mutant showed dramatically decreased levels of acylation when incubated with malonyl-CoA (see **Supplementary Figure S6**), and was omitted from further assays. The catalytic Ser706 and His808 residues are highlighted in magenta.

a). b).

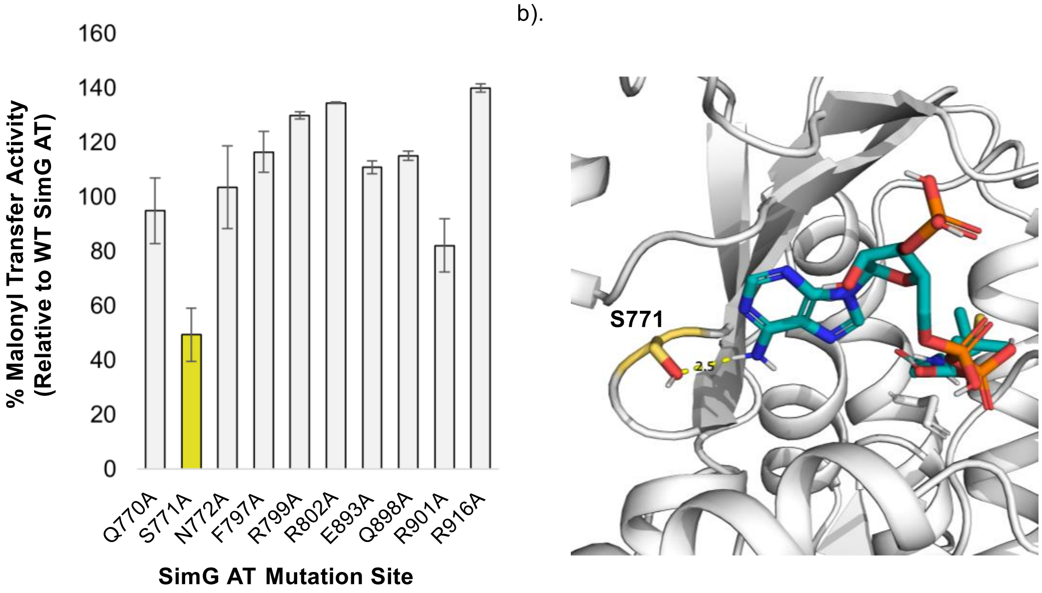

Supplementary Figure S6 | Acylation of SimG AT domain X→Ala mutants with malonyl-CoA. a). Bar chart showing percentage acylation of the X→Ala mutants generated for SimG AT domain, relative to wild-type SimG AT domain acylation. b). Alignment of the SimG AT domain AlphaFold 3 model with the *E. coli* FAS AT domain crystal structure with coenzyme A bound (PDB: 2G2Z). The proximity of Ser771 to the coenzyme A substrate likely provides a hydrogen bonding interaction which promotes acylation of the AT domain.

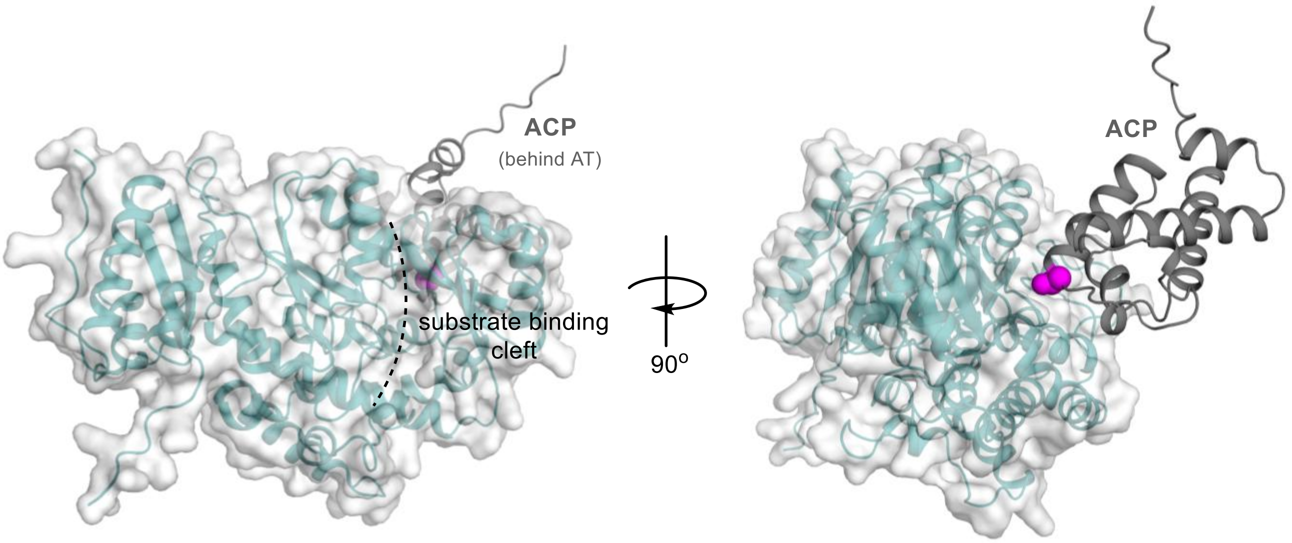

Supplementary Figure S7 | AlphaFold of SimG AT:ACP complex. Predicted of the SimG AT:ACP complex using AlphaFold. The ACP domain is placed behind the substrate binding cleft (indicated by a dashed line) of the AT domain. The site of Ppant attachment (Ser2514) is highlighted in magenta.

a). b).

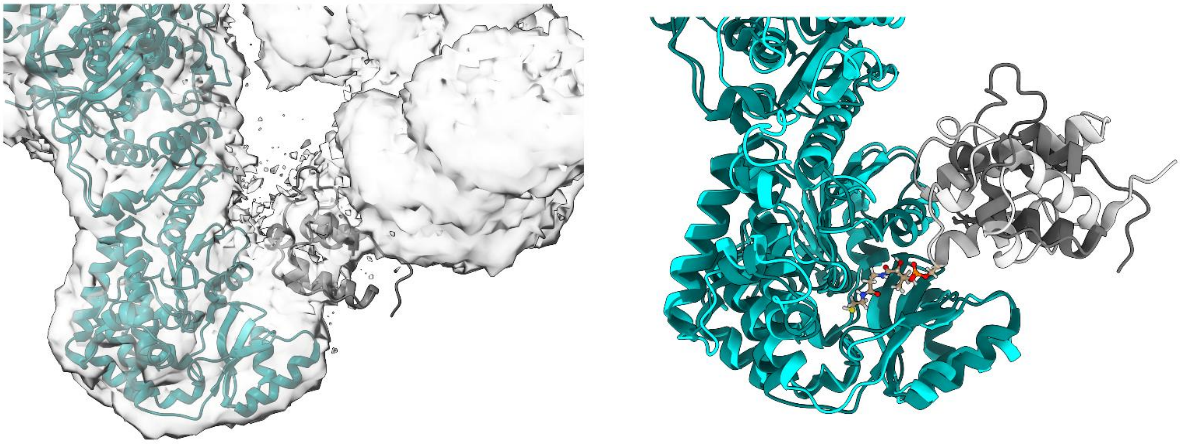

Supplementary Figure S8 | Comparison of docked SimG AT:ACP complex with mFAS cryo-EM density map. a). Overlay of the cryo-EM map (EMDB-43337) with atomic model of hFASN-NADPH state 1. MAT is indicated in teal. ACP fitted in partial density is indicated in dark grey. b). Overlay of the atomic model from a) with a representative conformation for the SimG AT-ACP complex determined in this work. AT is indicated in cyan. ACP is indicated in light grey. Post translationally modified Ser on ACP in the cryo-EM model is rendered with grey atoms. Loaded Ppant in our complex is also shown.

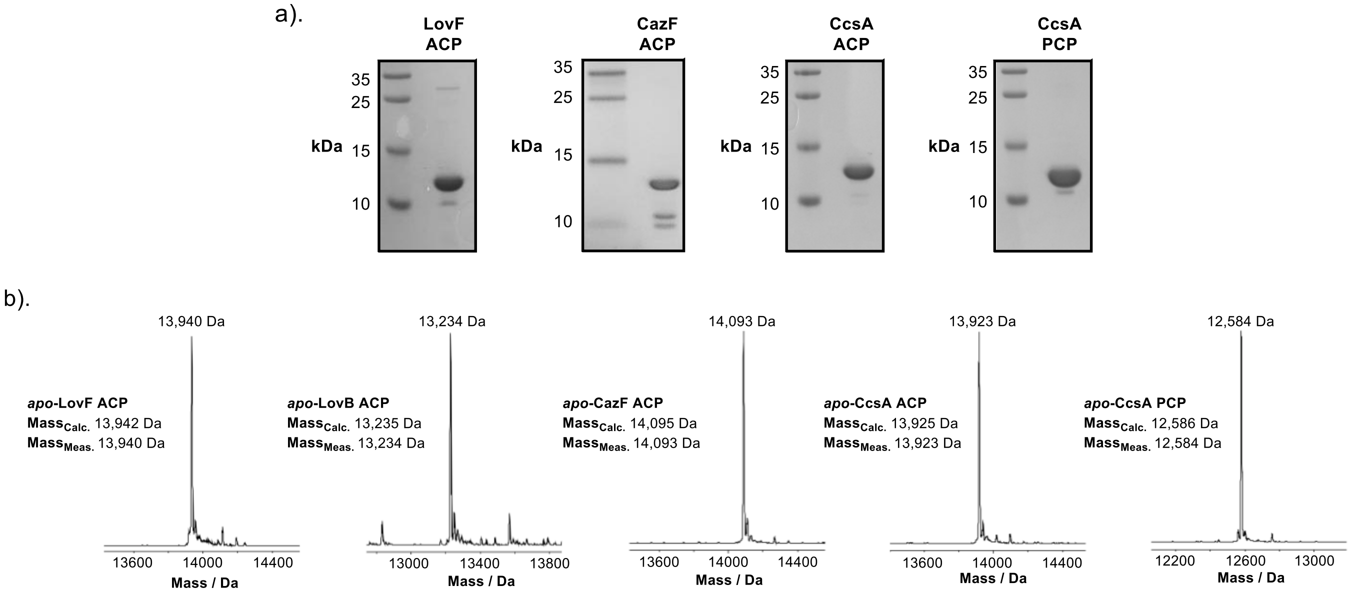

Supplementary Figure S9 | SDS-PAGE and mass spectrometry analysis of purified recombinant proteins. a). 15 % SDS-PAGE gel containing (from left to right): LovF ACP domain (~14 kDa), CazF ACP domain (~14 kDa), CcsA ACP domain (~14 kDa), and CcsA PCP domain (~13 kDa). b). Deconvoluted mass spectra of purified ACP / PCP domains with calculated and observed masses detailed. The mass spectrum of LovB ACP is shown as this is a modified construct from the previously reported construct, which contains additional His residues in the tag to improve purification.

a).

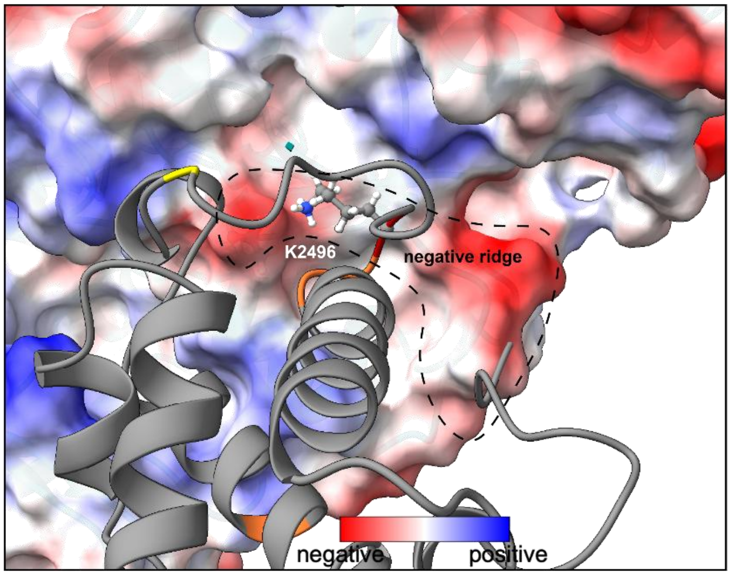

b).

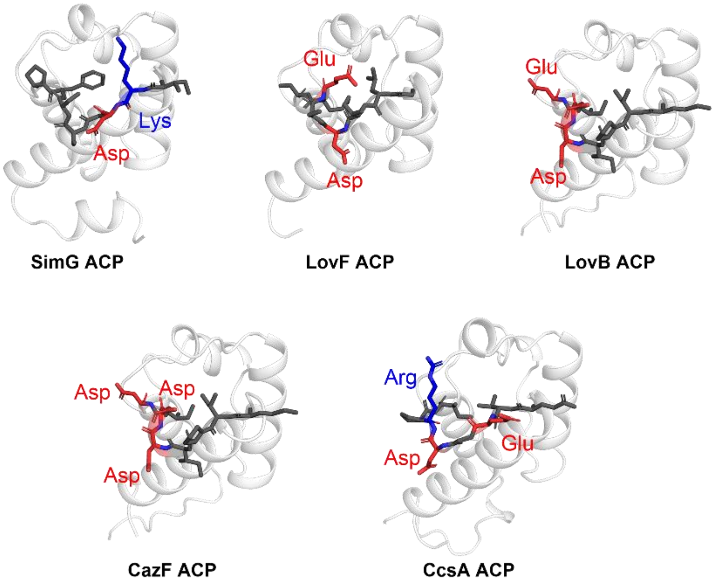

Supplementary Figure S10 | Electrostatic interactions between SimG AT and cognate / non-cognate ACP domains. a). Representative frame of the SimG ACP:AT complex following MD simulations (as shown in **Fig. 4a**). The surface electrostatics of the AT domain are displayed and the critical K2496 residue on the ACP domain is shown as sticks. The negatively charged ridge can be sampled by a positively charged residue (Lys / Arg) in the Loop I region. b). AlphaFold structural models of ACP domains used in malonyl transfer assays. The first 8 residues of the Loop I region are displayed as sticks, and negatively / positively charged residues are coloured red and blue, respectively. The presence of a positively charged residue in SimG ACP (cognate) and CcsA ACP (non-cognate) correlates with highest levels of malonyl transfer (**Fig. 5b**).

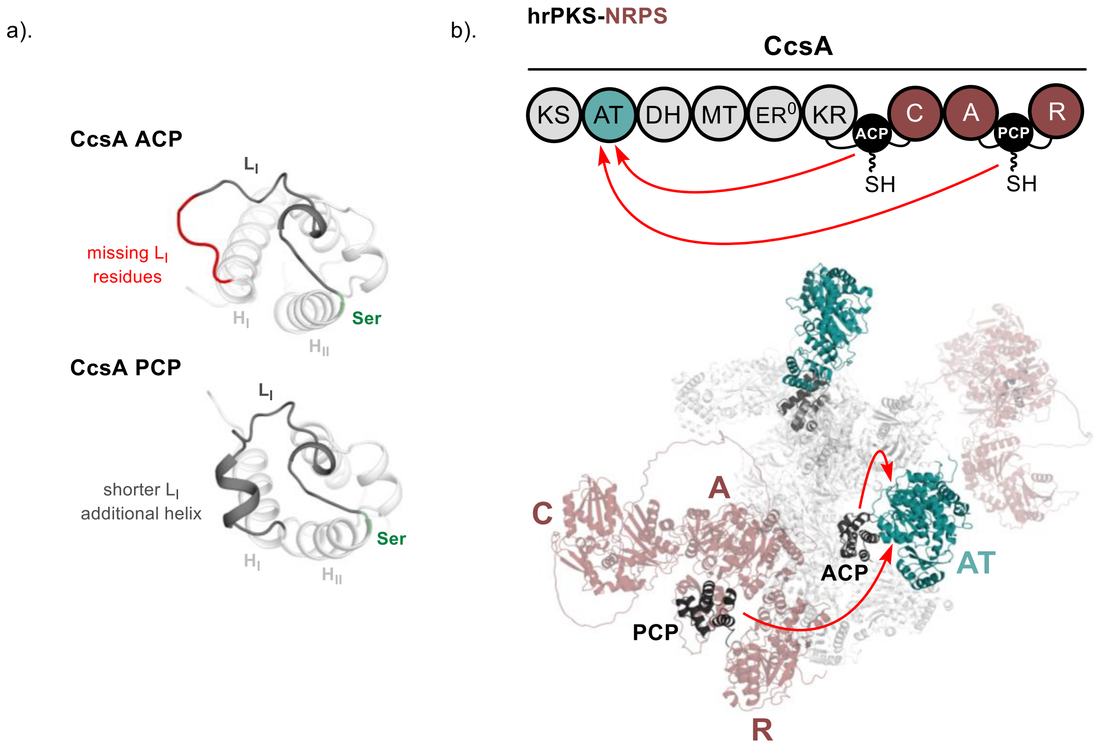

Supplementary Figure S11 | Structural differences and accessibility of carrier protein domains in PKS-NRPS hybrids. a). Structural comparison of AlphaFold models for CcsA ACP (*top*) and PCP (*bottom*) domains. The L_I region is highlighted in dark grey, and the residues missing from CcsA PCP are indicated in red. The site of Ppant attachment is highlighted in green. b). Domain arrangement (*top*) and AlphaFold model (*bottom*) of the entire CcsA protein. The protein was assembled into a dimer by structural alignment of two copies with the LovB hrPKS (PDB: 7CPX).[1] Whilst the flexible linkers may allow the PCP domain to access the AT domain, a combination of improved transacylation for the ACP domain (**Fig. 5b**), and engagement with the A, C and R domains of the NRPS region, likely mean that the PCP domain is not loaded with malonyl during catalysis.

2. Methods

2.1. Molecular cloning and mutagenesis.

The cloning of SimG AT and SimG ACP plasmids has been reported previously.[2] The plasmids for LovF ACP, LovB ACP, CazF ACP, CcsA ACP and CcsA PCP were gene synthesised (Epoch Life Sciences) and subcloned into pET24a with a custom $pHis_8$ tag (see **Section 3.1**). All SimG ACP and AT mutants were constructed using the Q5 site directed mutagenesis kit (NEB), using primers detailed in **Tables S1** and **S3** introducing a X→Ala mutation in each case. The resulting PCR products were processed according to the manufactures protocol, and resulting plasmids sequenced to verify correct mutation.

2.2. Protein overexpression and purification.

Overexpression and purification of SimG AT and ACP domains has been reported previously.[2] The same procedure was applied to all SimG ACP X→Ala mutants and non-cognate ACP domains (LovF ACP, LovB ACP, CazF ACP, CcsA ACP and CcsA PCP) used in this study. Briefly, an aliquot of chemically competent *E. coli* BL21 Star (DE3) cells (50 µL) was transformed with relevant plasmid DNA. Transformed cells were plated onto LB agar plates containing kanamycin (50 µg/mL) and incubated overnight at 37˚C. A single colony was picked and used to inoculate LB media (10 mL) containing kanamycin (50 µg/mL) and incubated overnight at 37˚C with shaking (180 rpm). The overnight culture was used to inoculate a flask of LB media (1 L) with kanamycin (50 µg/mL) which was left to grow at 37˚C with shaking (180 rpm) until an OD_{600} of 0.7 - 1.0 was reached. Protein expression was induced by the addition of IPTG (250 µM) and incubated at 15 ˚C with shaking (180 rpm) overnight.

Cells were centrifuged (4000 rpm, 15 minutes, 4 ˚C) and resuspended in loading buffer (20 mM Tris HCl, 100 mM NaCl, 20 mM imidazole, pH 8.0). The cells were lysed by high pressure (20 psi) cell disruption and centrifuged (17,000 rpm, 30 minutes, 4˚C) to pellet the insoluble cell components. The cell lysate was filtered through a 0.45 µm syringe filter and loaded onto a 1 mL HiTrap™ Nickel FastFlow column. Following a wash with loading buffer (10 mL), the protein was eluted from the column with buffers of increasing imidazole concentrations (5 mL of 50 mM, 3 mL of 100 mM, 3 mL of 200 mM, 3 mL of 300 mM). Fractions were checked for the protein of interest by SDS-PAGE and those containing protein were concentrated and exchanged into storage buffer (20 mM Tris HCl, 100 mM NaCl, pH 7.6) using an appropriate size Vivaspin centrifugal concentrator (4000 rpm, 4 ˚C). Once concentrated to approx. 1 mL, 50 µL aliquots of protein were flash frozen in liquid N_2 and stored at -80 ˚C.

2.3. Conversion of *apo*- to *holo*-ACP / PCP domains.

All *apo*-ACP / PCP (200 µM) species (including X→Ala mutants) were converted to their *holo*-ACP / PCP form by incubation in storage buffer (20 mM Tris base, 100 mM NaCl, pH 7.6) supplemented with 10 mM $MgCl_2$, 2 µM Sfp, and 1 mM CoA in a total of 50 µL for 30 minutes at 25 ˚C. Samples were diluted 10-fold with milliQ H_2O and analysed by UHPLC-ESI-Q-TOF-MS for successful conversion.

2.4. SimG AT transacylation time-course.

SimG *holo*-ACP domain (50 µM) was incubated with SimG AT domain (1 µM) and malonyl-CoA (250 µM) in storage buffer (20 mM Tris base, 100 mM NaCl, pH 7.6) made up to a final volume of a final volume of 100 µL at 25 ºC. The reaction was quenched with formic acid (1 % v/v final) at intervals between 0.5 → 20 mins following AT domain incubation, before dilution with milliQ H_2O and analysis by UHPLC-ESI-Q-TOF-MS. Raw mass spectra were subjected to deconvolution (Bruker MaxEnt), taking an average of all charge states. Using the raw peak intensities for *holo*-ACP and acyl-ACP, percentage conversion at a given time point was calculated using the following equation:

$$\%\ acyl\text{-ACP} = \frac{Inten._{acyl\text{-}ACP}}{(Inten._{holo\text{-ACP}} + Inten._{\text{acyl-ACP}})}$$

All time-point measurements were conducted in triplicate and average values were plotted. The average acylation value at the 2 min time point was 58.3 ± 1.44 %.

2.5. Assessment of transacylation activity for SimG ACP X→Ala mutant library.

Each SimG ACP domain X→Ala mutant (50 µM) was incubated with SimG AT domain (1 µM) and malonyl-CoA (250 µM) in storage buffer (20 mM Tris base, 100 mM NaCl, pH 7.6) made up to a final volume of a final volume of 50 µL at 25 ºC. The reaction was quenched with formic acid (1 % v/v final) after 2 min, and samples were diluted 5-fold with milliQ H_2O for analysis by UHPLC-ESI-Q-TOF-MS. Percentage conversion to the malonyl-ACP domain species for each mutant was calculated as outlined in **Section 2.4**. This conversion was made relative to the wild-type ACP conversion using the following equation:

$$SimG\ ACP\ (X \rightarrow Ala)\ activity = \left(\frac{\%\ acyl\text{-ACP (X→Ala } mutant\text{)}}{\text{58.3 (WT } activity\text{)}}\right) \times 100$$

2.6. UHPLC-ESI-Q-TOF-MS analysis of SimG ACP.

All transacylation assays were analysed on a Bruker MaXis II ESI-Q-TOF-MS connected to a Dionex 3000 RS UHPLC fitted with an ACE C4-300 RP column (100 x 2.1 mm, 5 µm, 30 °C). The column was eluted with a linear gradient of 5 – 100% MeCN containing 0.1 % formic acid over 30 min. The mass spectrometer was operated in positive ion mode with a scan range of 200 – 3000 *m/z*. Source conditions were: end plate offset at −500 V; capillary at −4500 V; nebulizer gas (N_2) at 1.8 bar; dry gas (N_2) at 9.0 L min^{-1} ; dry temperature at 200 °C. Ion transfer conditions were: ion funnel RF at 400 Vpp; multiple RF at 200 Vpp; quadrupole low mass at 200 *m/z*; collision RF at 2000 Vpp; transfer time at 110.0 µs; pre-pulse storage time at 10.0 µs. Measured masses for the intact *apo*-ACP and AT species are displayed in **Table S2** and **S4**.

2.7. Circular dichroism analyses of SimG ACP and X→Ala mutants.

Circular dichroism spectra of SimG ACP and associated mutants were acquired on a JASCO J-1500 spectropolarimeter, using a 1 mm path length quartz cuvette. Spectra were acquired between 190 and 260 nm at room temperature. Before analysis protein samples were exchanged into CD buffer (0.5 mM Tris HCl, 5 mM NaCl, pH 7.4) and adjusted to a final concentration of 0.1 mg/mL.

2.8. AlphaFold 3 model generation, residue-guided protein docking and molecular dynamics.

2.8.1. AlphaFold 3 model generation of SimG AT and SimG ACP domains.

Structural models of SimG AT and ACP domains were generated using AlphaFold 3.[3] Input sequences for each domain did not include His-Tag / non-native amino acids.

2.8.2. Residue-guided protein docking of the SimG AT:ACP complex.

Docking simulations between the SimG ACP and AT domain were performed using the High Ambiguity Driven DOCKing (HADDOCK 2.4) webserver.[4] Simulations were conducted using structural models of each domain, as outlined in **Section 2.8.1**. Residues identified from alanine scanning experiments were defined as 'active' in the docking simulation, and Ser2514 on the ACP domain defined as 'passive' as it is the Ppant attachment site. Results from the docking were evaluated for shape / charge complementarity and positioning of Ser2514 with respect to the substrate binding cleft. A structure from the highest-scoring docking solution cluster was taken forward for molecular dynamics.

2.8.3. Molecular dynamic simulations of SimG holo-ACP:malonyl-AT complex.

The co-ordinates for simulation of the SimG AT:ACP complex were taken from the first model of the highest scoring cluster from the docking simulation (see **Section 2.8.2**). A malonate was manually attached to Ser2514 using previous malonyl-AT structures as a guide[5,6], and the Ppant arm was placed manually in virtual reality using ChimeraX.[7–9] Molecular dynamics (MD) simulations were performed using the AMBER ff19SB force field.[10] Ppant and *O*-malonyl-serine were parametrised using ANTECHAMBER[11] and GAFF2 force field with ABCG2 charge model.[12] The resulting structure was neutralised with Na^+ ions and solvated with OPCBOX water, such that no atom belonging to the complex was less than 10 Å from any box edge, using the LEaP module.[13] Additional Na^+ and Cl^- ions were added to obtain 100 final salt concentration. MD heating, equilibration, and production steps

were performed using the GPU accelerated AMBER24 software[14] on an HPC cluster equipped with Nvidia L40 graphics cards. Simulation used the SHAKE algorithm[15] to constrain all protein bonds involving a hydrogen atom; a 2.0 fs time-step was used in these simulations. Long-range electrostatics were calculated using the Particle Mesh Ewald (PME) method with a 12.0 Å cut-off.[16] PME was used for nonbonded interactions. In all simulations, the Langevin thermostat (γ = 2.0 ps^{-1}) was used to maintain temperature control.[17] The solvated protein was then equilibrated by carrying out a short minimization, 50 ps of heating and 50 ps of density equilibration with weak restraints on the protein followed by 500 ps of constant pressure equilibration at 300 K. After a two-step minimization process, in which solvent molecules were allowed to relax before the entire system was minimized, the system was slowly heated to 300 K over 0.1 ns in a canonical ensemble (NVT) simulation, then equilibrated for 2 ns by performing isothermal-isobaric (NPT) simulations at 300K using a Berendsen barostat.[18] Three independent repeats of 250 ns production using classic approach (with no acceleration) run were performed with simulation frames written every 20 ps for analysis. Additional 200 ns accelerated MD (aMD) was run from a restart point of the third repeat of the classical MD. For aMD frames were written every 20 ps. The aMD modification of the potential were defined as:

$$V(r)^* = V(r) + \Delta V(r)$$

$$\Delta V(r) = \frac{(E_P - V(r))^2}{(\alpha_P + E_P - V(r)} + \frac{(E_D - V(r))^2}{(\alpha_D + E_D - V_D(r)}$$

where $V(r)$ is the normal potential and $V_D(r)$ is the normal torsion potential, with E_p = -400203.9002 kcal mol^{-1}; α_p = 27345.8 mol^{-1}; E_D = 4831.4334 kcal mol^{-1}; α_D = 385.7 kcal mol^{-1} (estimated from the cMD simulation, see Amber manual). For all the classic and accelerated simulations, long-range electrostatics were calculated using the PME method with 8.0 Å and 10.0 Å cutoffs, respectively. A combination of CCPTRAJ[19], Chimera[20] and ChimeraX[21] were used for analysis of the trajectories. ChimeraX was used throughout the course to prepare and visualize the structures.

The repeats of 250 ns cMD trajectories and one repeat of the 200 ns aMD trajectory stripped of water and ions with frames every 20 ns are available for download at https://doi.org/10.5281/zenodo.17272938.

3. Sequences, Tables and Accession Numbers

3.1. Amino acid sequences

Amino acid sequences for SimG hrPKS, SimG AT domain and SimG ACP domain have been reported previously.[2]

pHis$_8$_LovF ACP (*Aspergillus terreus*, Q9Y7D5)

```
MKHHHHHHHH GGLVPRGSHG S-
NHGALSLTPA EDDNLHARLN RAISQQESIA VIMEAMSCKL ISMFGLTDSE MSATQTLAGI
GVDSLVAIEL RNWITAKFNV DISVFELMEG RTIAKVAEVV LQRYKA-
```

MW (including His-tag) = 13,941.94 Da
Ppant attachment site highlighted in Cyan.

pHis$_8$_LovB ACP* (*Aspergillus terreus*, Q9Y7D5)

```
MKHHHHHHHH GGLVPRGSHG S-
GEGAAGSKGS VKEQLLQATN LDQVRQIVID GLSAKLQVTL QIPDGESVHP TIPLIDQGVD
SLGAVTVGTW FSKQLYLDLP LLKVLGGASI TDLANEAAAR LPPSS-
```

MW (including His-tag) = 13,234.99 Da
Ppant attachment site highlighted in Cyan.
* core amino acid sequence based on previously published LovB ACP construct.[22]

pHis$_8$_CazF ACP (*Chaetomium globosum*, A0A0K0MCJ4)

```
MKHHHHHHHH GGYVPRGSHG S-
GAQAAGQQLG DADVKPLSAQ LQEAGSRDEA TRLVGDAIAS KLGDIFMIPI DDIDLAKSPA
LHGVDSLVAV ELRNMLMLQA AADISIFSIM QSASLGALAS DVVAKSSHVE IAAGA
```

MW (including His-tag) = 14,094.91 Da
Ppant attachment site highlighted in Cyan.

pHis$_8$_CcsA ACP (*Aspergillus clavatus*, A1CLY8)

```
MKHHHHHHHH GGLVPRGSHG S-
STSAAPVKVR LAEASSSADI YDIISDAFVT KLKTSLQVEG DRPIVDLTAD TLGIDSLVAV
DIRSWFIKEL QVEIPVLKIL SGATVGEMVT QAQELLPKEL TPNLDPNA
```

MW (including His-tag) = 13,924.85 Da
Ppant attachment site highlighted in Cyan.

pHis$_8$_CcsA PCP (*Aspergillus clavatus*, A1CLY8)

```
MKHHHHHHHH GGLVPRGSHG S-
ADTGTDESPS MAKMRDVWAT VIPQEVLAHF ELGPASNFFQ VGGDSMLLVR LQTEINKVFG
TSISLFQLFD ASSLTGMVSL IDHSESTSQR SEVD
```

MW (including His-tag) = 12,586.04 Da
Ppant attachment site highlighted in Cyan.

3.2. Supplementary Tables

Table S1. Primers for SimG ACP domain X →Ala mutants.

SimG ACP Mutant	Primers For / Rev (5'→3')	Annealing Temperature (°C)
S2474A	Forward: CGCCGAGCCAGCAGAGGAGAAGAA<br>Reverse: GCCAGGACGACCTTGAGC	69
E2476A	Forward: CGAGCCATCTGCGGAGAAGAAGACC<br>Reverse: GCGGCCAGGACGACCTTG	72
T2479A	Forward: GGAGAAGAAGGCGGAGATCATCGCCAAGGC<br>Reverse: TCAGATGGCTCGGCGGCC	72
E2480A	Forward: GAAGAAGACCGCGATCATCGCCAAG<br>Reverse: TCCTCAGATGGCTCGGCG	67
T2489A	Forward: CCTCGCCGGCGCACTCGGGAACT<br>Reverse: GCCTTGGCGATGATCTCGG	69
N2492A	Forward: CACTCTCGGGGCGTTCCTCATCAAAGACGGGAGCAGCTTCCC<br>Reverse: CCGGCGAGGGCCTTGGCG	72
F2493A	Forward: TCTCGGGAACGCGCTCATCAAAGACGGGAGCAGCTTC<br>Reverse: GTGCCGGCGAGGGCCTTG	72
L2494A	Forward: CGGGAACTTCGCGATCAAAGACGGGAGCAGCTTCCC<br>Reverse: AGAGTGCCGGCGAGGGCC	72
I2495A	Forward: GAACTTCCTCGCGAAAGACGGGAGCAGCTTCCCAC<br>Reverse: CCGAGAGTGCCGGCGAGG	71
K2496A	Forward: CTTCCTCATCGCGGACGGGAGCAGCTTCCCAC<br>Reverse: TTCCCGAGAGTGCCGGCG	70
S2499A	Forward: CAAAGACGGGGCGAGCTTCCCACTC<br>Reverse: ATGAGGAAGTTCCCGAGAG	60
S2500A	Forward: AGACGGGAGCGCGTTCCCACTCG<br>Reverse: TTGATGAGGAAGTTCCCG	60
K2505A	Forward: CCCACTCGACGCACCCCTCAAGATG<br>Reverse: AAGCTGCTCCCGTCTTTG	62
K2508A	Forward: CAAGCCCCTCGCAATGCTCGGCATG<br>Reverse: TCGAGTGGGAAGCTGCTC	66
M2518A	Forward: TTTGATCGCCGCAGAGGTGCGGAAC<br>Reverse: GAATCCATGCCGAGCATC	62
E2519A	Forward: GATCGCCATGGCAGTGCGGAACT<br>Reverse: AAAGAATCCATGCCGAGC	63
R2521A	Forward: CATGGAGGTGGCAAACTGGATCC<br>Reverse: GCGATCAAAGAATCCATG	57
N2522A	Forward: GGAGGTGCGGGCATGGATCCGAC<br>Reverse: ATGGCGATCAAAGAATCC	60
R2525A	Forward: GAACTGGATCGCACAGAACATCGGCGC<br>Reverse: CGCACCTCCATGGCGATC	67
Q2526A	Forward: CTGGATCCGAGCAAACATCGGCGCCGAGACAAG<br>Reverse: TTCCGCACCTCCATGGCG	68
E2531A	Forward: CATCGGCGCCGCAACAAGCACGT<br>Reverse: TTCTGTCGGATCCAGTTCCG	67
T2532A	Forward: CGGCGCCGAGGCAAGCACGTTCA<br>Reverse: ATGTTCTGTCGGATCCAGTTCCGCAC	72
S2533A	Forward: CGCCGAGACAGCAACGTTCACCG<br>Reverse: CCGATGTTCTGTCGGATC	60
T2534A	Forward: CGAGACAAGCGCATTCACCGTGC<br>Reverse: GCGCCGATGTTCTGTCGG	67
F2535A	Forward: GACAAGCACGGCAACCGTGCTACAGAG<br>Reverse: TCGGCGCCGATGTTCTGT	66
T2536A	Forward: AAGCACGTTCGCAGTGCTACAGA<br>Reverse: GTCTCGGCGCCGATGTTC	65
Q2539A	Forward: CACCGTGCTAGCAAGCTCTTCCTTCATGCATCTG<br>Reverse: AACGTGCTTGTCTCGGCG	65
S2540A	Forward: CGTGCTACAGGCATCTTCCTTCATGCATC<br>Reverse: GTGAACGTGCTTGTCTCG	60
S2541A	Forward: GCTACAGAGCGCATCCTTCATGC<br>Reverse: ACGGTGAACGTGCTTGTC	63
E2549A	Forward: TCTGGCCGGCGCAATCAGGGCGG<br>Reverse: TGCATGAAGGAAGAGCTCTGTAGCAC	72
R2551A	Forward: CGGCGAGATCGCAGCGGCGATGA<br>Reverse: GCCAGATGCATGAAGGAAG	64
E2560A	Forward: GGCGAGCGAGGCATGAAAGCTTGCGG<br>Reverse: GCATTCATCGCCGCCCTG	68

Table S2. Measured masses of SimG ACP domain X →Ala mutants.

SimG ACP Mutant	Calculated Mass (Da)	Measured Mass (Da)
S2474A	13607.56	13607.50
E2476A	13565.52	13564.60
T2479A	13593.53	13591.73
E2480A	13565.52	13564.85
T2489A	13593.53	13593.56
N2492A	13580.53	13579.48
F2493A	13547.46	13547.45
L2494A	13581.48	13580.63
I2495A	13581.48	13580.81
K2496A	13566.46	13565.62
S2499A	13607.56	13606.69
S2500A	13607.56	13606.60
K2505A	13566.46	13566.56
K2508A	13566.46	13565.88
M2518A	13563.45	13562.63
E2519A	13565.52	13564.00
R2521A	13538.45	13537.69
N2522A	13580.53	13579.88
R2525A	13538.45	13537.69
Q2526A	13566.51	13565.63
E2531A	13565.52	13564.69
T2532A	13593.53	13592.63
S2533A	13607.56	13606.88
T2534A	13593.53	13592.81
F2535A	13547.46	13546.69
T2536A	13593.53	13592.63
Q2539A	13566.51	13566.56
S2540A	13607.56	13605.65
S2541A	13607.56	13606.69
E2549A	13565.52	13564.56
R2551A	13538.45	13537.69
E2560A	13565.52	13564.69

Table S3. Primers for SimG AT domain X →Ala mutants

SimG AT Mutant	Primers For / Rev (5'→3')	Annealing Temperature (°C)
Q770A	Forward: CGAGAACAGTGCAAGCAACGTCACGCTGTCGG<br>Reverse: CAGGCGATGTTGACCCCC	66
S771A	Forward: GAACAGTCAGGCAAACGTCACGCTGTCGG<br>Reverse: TCGCAGGCGATGTTGACC	64
N772A	Forward: CAGTCAGAGCGCAGTCACGCTGTCG<br>Reverse: TTCTCGAGGCGATGTTG	61
F797A	Forward: ACCGGGGGTCGCAGCTCGTTTGC<br>Reverse: CTCTCGGCCTTGAGCGTC	65
R799A	Forward: GGTCTTTGCTGCATTGCTCCGGGTAGAGAAGGC<br>Reverse: CCCGGTCTCTCGGCCTTG	67
R802A	Forward: TCGTTTGCTCGCAGTAGAGAAGGCGTATCACTC<br>Reverse: GCAAAGACCCCCGGTCTC	66
E893A	Forward: TCCCGCCCTTGCAGGACCAGTCG<br>Reverse: TGAGGTCCCAACTCGATTAGGAC	69
Q898A	Forward: ACCAGTCGGCGCAATTCTCCGAGACCTGG<br>Reverse: CCTTCAAGGGCGGGATGA	65
R901A	Forward: CCAGATTCTCGCAGACCTGGGTCG<br>Reverse: CCGACTGGTCCTTCAAGG	63
R916A	Forward: GACCTGTATCGCAGGCAGCGAGTG<br>Reverse: CCAAGGTAGATGTCGTCTG	60

Table S4. Measured masses of SimG AT domain X →Ala mutants.

SimG AT Mutant	Calculated Mass (Da)	Measured Mass (Da)
Q770A	51104.67	51101.44
S771A	51145.67	51142.50
N772A	51118.70	51117.38
F797A	51085.62	51083.59
R799A	51076.61	51074.66
R802A	51076.61	51074.80
E893A	51103.68	51100.53
Q898A	51104.67	51101.84
R901A	51076.61	51073.39
R916A	51076.61	49075.00*

* degradation observed, but full activity in assays.

4. References

1 J. Wang, J. Liang, L. Chen, W. Zhang, L. Kong, C. Peng, C. Su, Y. Tang, Z. Deng and Z. Wang, *Nat. Commun.*, 2021, **12**, 867.
2 M. E. Foran, N. B. Auckloo, Y. T. C. Ho, S. Liu, Y. Hai and M. Jenner, *Chem. Sci.*, 2025, **16**, 13173–13182.
3 J. Abramson, J. Adler, J. Dunger, R. Evans, T. Green, A. Pritzel, O. Ronneberger, L. Willmore, A. J. Ballard, J. Bambrick, S. W. Bodenstein, D. A. Evans, C.-C. Hung, M. O'Neill, D. Reiman, K. Tunyasuvunakool, Z. Wu, A. Žemgulytė, E. Arvaniti, C. Beattie, O. Bertolli, A. Bridgland, A. Cherepanov, M. Congreve, A. I. Cowen-Rivers, A. Cowie, M. Figurnov, F. B. Fuchs, H. Gladman, R. Jain, Y. A. Khan, C. M. R. Low, K. Perlin, A. Potapenko, P. Savy, S. Singh, A. Stecula, A. Thillaisundaram, C. Tong, S. Yakneen, E. D. Zhong, M. Zielinski, A. Žídek, V. Bapst, P. Kohli, M. Jaderberg, D. Hassabis and J. M. Jumper, *Nature*, 2024, **630**, 493–500.
4 R. V. Honorato, M. E. Trellet, B. Jiménez-García, J. J. Schaarschmidt, M. Giulini, V. Reys, P. I. Koukos, J. P. G. L. M. Rodrigues, E. Karaca, G. C. P. van Zundert, J. Roel-Touris, C. W. van Noort, Z. Jandová, A. S. J. Melquiond and A. M. J. J. Bonvin, *Nat. Protoc.*, 2024, **19**, 3219–3241.
5 C. Oefner, H. Schulz, A. D'Arcy and G. E. Dale, *Acta Crystallogr. D Biol. Crystallogr.*, 2006, **62**, 613–618.
6 C. W. Liew, M. Nilsson, M. W. Chen, H. Sun, T. Cornvik, Z.-X. Liang and J. Lescar, *J. Biol. Chem.*, 2012, **287**, 23203–23215.
7 E. F. Pettersen, T. D. Goddard, C. C. Huang, E. C. Meng, G. S. Couch, T. I. Croll, J. H. Morris and T. E. Ferrin, *Protein Sci*, 2021, **30**, 70–82.
8 T. D. Goddard, C. C. Huang, E. C. Meng, E. F. Pettersen, G. S. Couch, J. H. Morris and T. E. Ferrin, *Protein Sci.*, 2018, **27**, 14–25.
9 T. D. Goddard, A. A. Brilliant, T. L. Skillman, S. Vergenz, J. Tyrwhitt-Drake, E. C. Meng and T. E. Ferrin, *J. Mol. Biol.*, 2018, **430**, 3982–3996.
10 C. Tian, K. Kasavajhala, K. A. A. Belfon, L. Raguette, H. Huang, A. N. Migues, J. Bickel, Y. Wang, J. Pincay, Q. Wu and C. Simmerling, *J. Chem. Theory Comput.*, 2020, **16**, 528–552.
11 J. Wang, W. Wang, P. A. Kollman and D. A. Case, *J. Mol. Graph Model*, 2006, **25**, 247–260.
12 X. He, V. H. Man, W. Yang, T.-S. Lee and J. Wang, *J. Chem. Phys.*, 2020, **153**, 114502.
13 C. E. A. F. Schafmeister, W. S. Ross and V. Romanovski, *LEap. University of California, San Francisco,* 1995.
14 D.A. Case, H.M. Aktulga, K. Belfon, I.Y. Ben-Shalom, J.T. Berryman, S.R. Brozell, F.S. Carvalho, D.S. Cerutti, T.E. Cheatham, III, G.A. Cisneros, V.W.D. Cruzeiro, T.A. Darden, N. Forouzesh, M. Ghazimirsaeed, G. Giambas¸u, T. Giese, M.K. Gilson, H. Gohlke, A.W. Goetz, J. Harris, Z. Huang, S. Izadi, S.A. Izmailov, K. Kasavajhala, M.C. Kaymak, I. Kolossva ry, A. Kovalenko, T. Kurtzman, T.S. Lee, P. Li, Z. Li, C. Lin, J. Liu, ´ T. Luchko, R. Luo, M. Machado, M. Manathunga, K.M. Merz, Y. Miao, O. Mikhailovskii, G. Monard, H. Nguyen, K.A. O'Hearn, A. Onufriev, F. Pan, S. Pantano, A. Rahnamoun, D.R. Roe, A. Roitberg, C. Sagui, S. Schott-Verdugo, A. Shajan, J. Shen, C.L. Simmerling, N.R. Skrynnikov, J. Smith, J. Swails, R.C. Walker, J. Wang, J. Wang, X. Wu, Y. Wu, Y. Xiong, Y. Xue, D.M. York, C. Zhao, Q. Zhu, and P.A. Kollman, *Amber 2025, University of California, San Francisco*, 2025.
15 J. P. Ryckaert, G. Ciccotti and H. J. C. Berendsen, *J. Comput. Phys.*, 1977, **23**, 327–341.
16 U. Essmann, L. Perera, M. L. Berkowitz, T. Darden, H. Lee and L. G. Pedersen, *J. Chem. Phys.*, 1995, **103**, 8577–8593.
17 R. J. Loncharich, B. R. Brooks and R. W. Pastor, *Biopolymers*, 1992, **32**, 523–535.
18 H. J. C. Berendsen, J. P. M. Postma, W. F. Van Gunsteren, A. Dinola and J. R. Haak, *J. Chem. Phys.*, 1984, **81**, 3684–3690.
19 D. R. Roe and T. E. I. Cheatham, *J. Chem. Theory Comput.*, 2013, **9**, 3084–3095.
20 E. F. Pettersen, T. D. Goddard, C. C. Huang, G. S. Couch, D. M. Greenblatt, E. C. Meng and T. E. Ferrin, *J. Comput. Chem.*, 2004, **25**, 1605–1612.
21 E. C. Meng, T. D. Goddard, E. F. Pettersen, G. S. Couch, Z. J. Pearson, J. H. Morris and T. E. Ferrin, *Protein Sci*, 2023, **32**, e4792.
22 S. M. Ma and Y. Tang, *FEBS J.*, 2007, **274**, 2854–2864.
